# High-Throughput Particle Concentration Using Complex Cross-Section Microchannels

**DOI:** 10.3390/mi11040440

**Published:** 2020-04-22

**Authors:** Asma Mihandoust, Sajad Razavi Bazaz, Nahid Maleki-Jirsaraei, Majid Alizadeh, Robert A. Taylor, Majid Ebrahimi Warkiani

**Affiliations:** 1 Complex Systems Laboratory, School of Physics-Chemistry, Department of Physics, Alzahra University, Tehran 1993893973, Iran; a.mihandoust@alzahra.ac.ir (A.M.); maleki@alzahra.ac.ir (N.M.-J.); 2 School of Biomedical Engineering, University of Technology Sydney, Sydney, NSW 2007, Australia; sajad.razavibazaz@student.uts.edu.au; 3 School of Paramedicine, Ilam University of Medical Science, Ilam 6939177143, Iran; alizadeh_majid@ymail.com; 4 School of Mechanical and Manufacturing Engineering, University of New South Wales, Sydney, NSW 2052, Australia; robert.taylor@unsw.edu.au; 5 Institute of Molecular Medicine, Sechenov University, 119991 Moscow, Russia

**Keywords:** Inertial microfluidics, complex cross-section, particle/cell concentrator

## Abstract

High throughput particle/cell concentration is crucial for a wide variety of biomedical, clinical, and environmental applications. In this work, we have proposed a passive spiral microfluidic concentrator with a complex cross-sectional shape, i.e., a combination of rectangle and trapezoid, for high separation efficiency and a confinement ratio less than 0.07. Particle focusing in our microfluidic system was observed in a single, tight focusing line, in which higher particle concentration is possible, as compared with simple rectangular or trapezoidal cross-sections with similar flow area. The sharper focusing stems from the confinement of Dean vortices in the trapezoidal region of the complex cross-section. To quantify this effect, we introduce a new parameter, complex focusing number or CFN, which is indicative of the enhancement of inertial focusing of particles in these channels. Three spiral microchannels with various widths of 400 µm, 500 µm, and 600 µm, with the corresponding CFNs of 4.3, 4.5, and 6, respectively, were used. The device with the total width of 600 µm was shown to have a separation efficiency of ~98%, and by recirculating, the output concentration of the sample was 500 times higher than the initial input. Finally, the investigation of results showed that the magnitude of CFN relies entirely on the microchannel geometry, and it is independent of the overall width of the channel cross-section. We envision that this concept of particle focusing through complex cross-sections will prove useful in paving the way towards more efficient inertial microfluidic devices.

## 1. Introduction

Concentrating cells and particles has long been regarded as an essential pre-treatment step in biomedical microanalysis, clinical diagnosis, and environmental applications [1,2]. Over the past two decades, microfluidic devices emerged as a means to increase control and sensitivity over traditional methods of concentrating particles [3]. Microfluidic particle/cell concentrators can be categorized as active and passive. Active techniques require the use of an external force field (e.g., dielectrophoresis, magnetophoresis, and acoustophoresis) for functionality [4,5]; however, they suffer from inherent limitations of low throughput and system complexity. This has limited the uptake of ‘active’ microfluidic devices. Passive techniques, on the other hand, rely solely on the hydrodynamic forces that naturally occur when flow passes through the microchannel’s geometry. Due to this simplicity, passive techniques have experienced massive research development (e.g., deterministic lateral displacement (DLD), pinched flow fractionation (PFF), and—as the relevant subject of this paper—inertial microfluidics) [6]. Inertial microfluidics enjoys a number of advantages, including less potential for clogging, relatively low fabrication cost, ease of operation, and high processing throughput [7,8,9,10]. To date though, only relatively simple cross-sectional geometries (i.e., circular, rectangular, and trapezoidal) have been used for the separation and filtration applications [11]. 

Inertial microfluidic devices work by moving randomly dispersed particles to certain equilibrium positions located between the wall and centerline of the microchannel, where a balance occurs for the inertial lift forces [12]. In square channels, particles focus at four equilibrium positions close to the center of each channel face. For rectangular cross-sectional shapes (e.g., those with high/low aspect ratio (AR) geometry), it is possible to reduce the number of particle equilibrium positions [13]. The number of particle focusing positions can also be reduced by adding contraction-expansion array (CEA) or curvature to the channel geometry [14,15]. In these designs, secondary flows induce yet another hydrodynamic force that can be tailored to balance the inertial lift forces more precisely and to accelerate the lateral migration of particles/cells across the channel [16]. In 2007, Seo et al. introduced a double-spiral designed microchannel that provided continuous and stable Dean vortices to help separate microparticles using this principle [17]. Since this seminal research, spiral microchannels have become the dominant design for focusing particles of different sizes at distinct equilibrium positions [18,19,20]. In 2014, Martel and Toner proposed a non-dimensional factor defined as the ratio of lift forces to Dean drag force, R_f_, to characterize the inertial focusing flows in curved channels [21]. This difference in lateral positions of particles with varying sizes is further amplified upon introducing a trapezoidal cross-section which is able to separate polymorphonuclear leukocytes (PMNs) and mononuclear leukocytes (MNLs) from diluted human blood with efficiency >80% [22]. Afterward, Martel et al. introduced a hydraulic resistance controlled micro-siphoning for a continuous concentration of cells [23]. In their work, cells were settled in the cell-rich fluid region at a flow rate of 500 µL/min, and the excess fluid was aspirated from the cell-free fluid region. Later on, Wang et al. utilized a passive inertial microfluidic device for concentrating cyanobacteria [24]. Although the small-sized cyanobacteria were isolated successfully, the flow rate is limited within the microscale due to the small channel cross-section. In 2018, Xiang et al. introduced an inertial microfluidic syringe cell concentrator which can efficiently isolate cells and remove the cell-free fluid [25]. They also have developed a multilayer spiral channel device for microalgae concentration and tumor cells isolation from large background fluids through vertically stacking multiplexing layers [26]. In spite of great performance achieved with these channels, they are still facing challenges in separating small particles with high throughput. In addition, in some cases, the requirement of assembling multilayer structures complicate the device fabrication and potentially enhance the cost and energy consumption.

To date, researchers have studied the effects of channel cross-section, curvature radius, and aspect ratio in spiral channels on particle lateral migration behavior. Little attention has been paid to optimize the threshold of the confinement ratio (CR) regarding the channel cross-sectional geometry. The $CR=a_{p}/D_{h}$ (where $a_{p}$ is the particle diameter and $D_{h}$ is the hydraulic diameter of the channel) is validated experimentally as a design parameter that can predict the focusing behavior of particles through microchannels [12]. From previous studies, there is a wide range of CR > 0.07 for successful inertial migration of particles resulting in a tight focusing band. Herein, we introduce a passive spiral microfluidic device with a complex cross-section for high-throughput particle concentration using the Dean drag force coupled with the inertial microfluidics phenomenon. We have shown our spiral microchannel with complex cross-sectional shape is capable of overcoming challenges associated with CR > 0.07 to achieve tight focusing bands. In most often, small channel cross-sections, which are restricted to $a_{p}/D_{h}>0.07$ to provide effective inertial lift forces, suffer from critically high fluidic resistance and require more power to pump the sample into the microfluidic device. Our approach can release this limitation. In this work, we altered the threshold of CR to a number less than 0.07. Particles were successfully focused on channels with complex cross-sectional shapes. We also investigated the particle migration behavior within three complex cross-sectional channels to find another effective parameter, i.e., focusing number of complex cross-section (CFN), to confine the focusing positions to particular areas. Our findings provide new insight into the fabricating inertial microchannel with complex cross-sections to be widely employed as a concentration tool in the areas of environmental analysis and disease diagnosis. 

## 2. Materials and Methods

### 2.1. Experimental Methods

#### 2.1.1. Device Fabrication 

**Figure 1:**
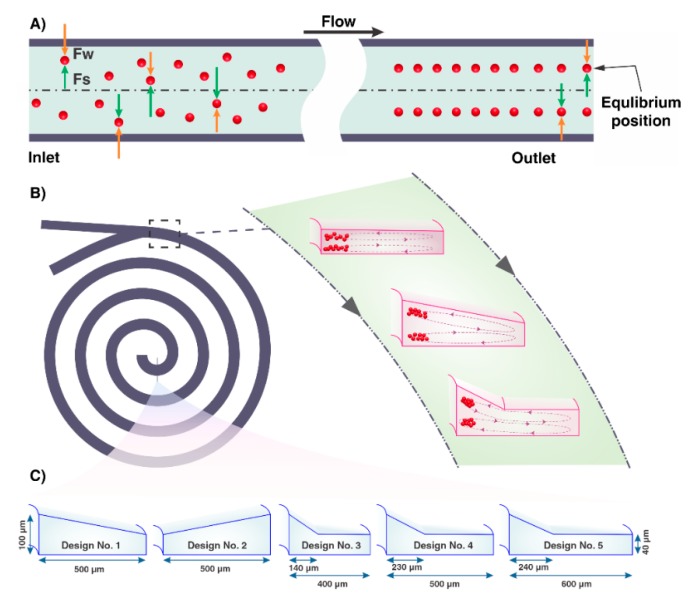
(**A**) The schematic illustration of particle focusing in straight rectangular microchannels. Particles that are initially dispersed will gradually migrate to their equilibrium positions and remain well-focused and stable due to the balance of opposing forces related to the shear gradient lift (*F_S_*) and wall interaction lift (*F_W_*) forces. (**B**) Schematic of spiral microchannels. Dean flow generates two counter-rotating vortices perpendicular to the main flow direction. (**C**) Schematic of a cross-sectional view of the channels used in this work.

Microchannels were fabricated in polydimethylsiloxane (PDMS, Sylgard 184, Dow Corning, Midland, MI, USA) using softlithography. Briefly, the channel mold, made by aluminum, was designed in SolidWorks (Dassault Systèmes SolidWorks Corporation, Waltham, MA, USA) and manufactured by the micro-milling technique to replicate microchannel features. Generally, milling systems consist of I. a worktable for positioning the workpiece, II. a cutting tool (most commonly an endmill), and III. an overhead spindle for securing and rotating the cutting tool. Modern mills employ computer numerical control (CNC) that automates the process to improve the repeatability and precision and reduce human error [27,28]. After the fabrication of mold, PDMS polymer mixed in a 10:1 ratio with the curing agent was casted on the aluminum master, degassed in a vacuum chamber, and baked in an oven for 2 h at 65 °C. Thereafter, PDMS was peeled off from the mold and bonded to a flat, thick PDMS layer using an Oxygen plasma unit (PDC-002, Harrick Plasma, Ossining, NY, USA). Finally, the fabricated chips were post backed for another 2 h at 65 °C. In addition, inlet and outlets were punched using a Uni-Core puncher (Sigma-Aldrich Co. LLC, SG, St. Louis, Mo, USA) prior to bonding. As can be seen from Figure 1, all spiral microchannels have 4.5 loops. Their cross-section varies from inward sloping trapezoid to outward sloping trapezoid and the complex cross-section combined of two parts; an outer part and an inner part. The outer part is rectangular, and the inner part is an inward sloping trapezoid. Additionally, the proportion of the width of the inner part (*w*_0_) to the total width of channel cross-section (*w*) defined as *w*_0_/*w*. The total width of channels was 600 µm, 500 µm, and 400 µm and the height of the inner and outer wall in all channels were the same and are 100 and 40 µm, respectively. 

#### 2.1.2. Experimental Operation 

Each fluorescent polystyrene microsphere with different sizes (4 µm and 6 µm, purchased from Fluoresbrite Microspheres, Polysciences Inc, City, Singapore) was diluted in the MACS buffer (~0.05 volume fraction) consisting of 1×phosphate buffered saline (PBS), and 2 mM EDTA supplemented with 0.5% bovine serum albumin (BSA) (Miltenyi Biotec, Bergisch Gladbach, Germany) to prevent nonspecific adhesion of microbeads to the tube and microchannel walls [29]. The prepared particle suspension was loaded into BD plastic 10 mL syringe which was then mounted onto a syringe pump. In order to precisely control the pumping process, a programmable syringe pump was used (Chemyx Fusion 200, Chemyx Inc., Stafford, TeX, USA). The sample fluid was driven through the microchannels at varying flow rates ranged from 0.5 to 3.0 mL/min. The trajectories of particle migration flowing in microchannels in various downstream were recorded using an inverted epifluorescence microscope (Olympus IX73 microscope, Olympus Inc., Shinjuku, Tokyo, Japan) equipped with a DP80 dual-chip charged coupled device (CCD) camera (Olympus Inc., Shinjuku, Tokyo, Japan). The exposure time and sensitivity were set to 50 ms and ISO 800-1600, respectively to ensure the fast shutter speed.

#### 2.1.3. Data Processing 

TechSmith Camtasia Studio (8.6.0) was used to record the screen while particles reach the steady-state conditions. The recording video files were rendered as a full-frame (i.e., uncompressed) in the format of AVI at 30 fps. Adobe photoshop (2015), OriginPro (2018), and ImageJ (1.52a) software were used for image processing. 

### 2.2. Simulation

In order to gain better insight through velocity profile distribution and secondary flow creation inside the microchannels, COMSOL Multiphysics 5.3a, a commercial software based on the finite element method was used [30]. At first, all geometries were designed via Solidworks, followed by exporting to Comsol. The Navier–Stokes and continuity equations (Equations (1) and (2)) were solved using a steady laminar flow module while incompressible and single-phase flow was assumed during the simulations.

(1) $$\rho \left(u\cdot \nabla \right)u=\nabla \cdot \left[-pI+\mu \left(\nabla u+{\left(\nabla \cdot u\right)}^{T}\right)\right]$$

(2) ρ∇.u=0

where u is the velocity vector, p is the pressure, ρ is the density, and μ is the dynamic viscosity. Normal inflow velocity was set as the inlet, while zero pressure was applied at the outlet. All channel walls were also assigned as no-slip velocity conditions. The density of 998 kg/m^3^ and dynamic viscosity of 0.00089 Pa·s was assumed for fluid properties inside the channel. Simulations were carried out using a remote desktop computer containing a 2 × 2.8 GHz Intel Xeon E5-2680 v2 (10 Cores) 25 MB L3 Cache 8 GT/s QPI (Max Turbo Freq. 3.6 GHz, Min 3.1 GHz), 256 GB 1866 MHz ECC DDR3-RAM (Quad Channel), and NVIDIA Quadro K2000 2 GB Graphics Card. 

To make sure that the results are the independence of the number of elements, various grid densities were tested, and it was found that mesh with the order of higher than 10^7^ was accurate enough to create stable hence reliable results. In addition, the iterative method with the solver of generalized minimal residual (GMRES) was used to solve the fluid domain of the current study. 

## 3. Results and Discussion 

### 3.1. Design Principle 

It has been clarified that the channel cross-section plays an essential role in particle manipulation. When flowing through a straight rectangular microchannel, initially random suspensions of particles reach certain equilibrium positions. In certain Re, these equilibrium positions are parallel to each other and the adjacent walls [31,32]. Spontaneous lateral migration of particles in Poiseuille flow and finite channel Re arises from a balance between dominant lift forces (*F_L_*), including shear-gradient-induced lift force (*F_S_*), and wall induced lift force (*F_W_*) which are orthogonal to the flow directions [33]. The schematic of inertial lift forces has been shown in Figure 1A. The shear-gradient-induced lift force occurs due to the curvature of the main velocity profile, pushing particles away from the channel center. The wall-induced lift force arises from the interaction between the particle and the walls of the channel, directing the particle away from the stationary wall of the channel and increases inversely with the normalized distance of the particle from the adjacent wall [34,35,36]. The mathematical relation describing the net magnitude of these two inertial lift forces is provided as follows [37]

(3) $$F_{L}=\frac{\rho U_{m}^{2}a_{p}^{4}}{D_{h}^{2}}f_{L}\left(Re_{c},X_{p}\right)$$

where $U_{m}$ is the maximum velocity of the flowing fluids, $a_{p}$ is the particle diameter, $f_{L}\left(Re_{c},X_{p}\right)$ is the dimensionless lift coefficient whose value and sign are dependent on the channel Re and the particle position within the channel cross-section ($X_{p}$), and $D_{h}$ is the hydraulic diameter of the channel.

Altering the geometry of the channel from straight to curve leads to further control of the number of equilibrium positions by inducing a secondary flow, illustrated as two symmetric-counter rotating vortices (Dean vortices) located in the top and bottom of the channel cross-section. These transverse vortices arise from the pressure gradient of fluid flow in the radial direction and are perpendicular to the primary flow direction across the channel cross-section [38,39]. The strength of Dean vortices is characterized by a dimensionless number called Dean number (De):

(4) $$De=Re\sqrt{\frac{D_{h}}{2R}}$$

where R is the radius of the channel [40]. The lateral Dean drag force $\left(F_{D}\right)$ induced by Dean vortices alter the focusing position of flowing particles, leading to the modification of focusing band depending on the position of the particles within vortices. The magnitude and direction of Dean drag forces are determined by the local vorticity field near the particle. The scaling of the Dean drag force can be assumed as follows [12].

(5) $$F_{D}\propto \rho U_{m}^{2}a_{p}D_{h}^{2}R^{-1}$$

Based on the balance of inertial lift forces (*F_L_*) and Dean drag force (*F_D_*), particles are focused at two discrete positions. 

The conceptual design of complex cross-sectional channels, a combination of trapezoid and rectangle shapes, is illustrated in Figure 1C. The trapezoid is located in the inner region of the channel cross-section, and its function is to isolate particles from carrier fluid. A rectangle, as a reservoir of the excess fluid, is an outer region of the channel cross-section driving particle-free fluid to the waste collection tube. All particles focused in the trapezoidal part of the complex cross-section form one narrow stream adjacent to the longer sidewall. The width assigned to the rectangular area of channel cross-section to the triangle height in the trapezoidal area (see the Supplementary Material and Figure S1) affects the degree of focusing band. Hence, we investigated the focusing behavior of particles, using a complex focusing number (CFN) for complex cross-sections, using Equation (6).

(6) $$CFN=\cot\alpha \frac{w_{R}}{w_{T}}$$

where α is the angle between the sloping side and the horizontal line, $w_{R}$ is the width of the trapezoidal region, and $w_{T}$ is the width of the rectangular region. Depending on the channel cross-section, there is a threshold of flow rate to exit particles from equilibrium positions. In our considered complex cross-sections, the amount of CFN affects this threshold, such that the higher the CFN, the higher the threshold, leading to enhance the total throughput of the device. 

**Figure 2:**
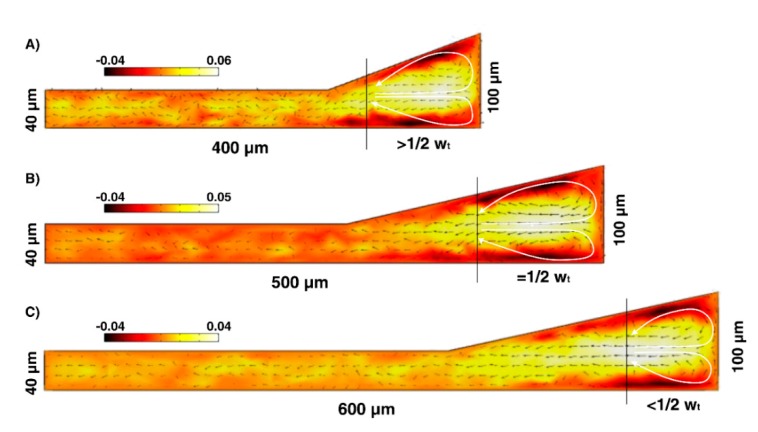
Simulation results indicating secondary flow in complex cross-sectional channels. The cross-section images illustrate that secondary vortices have been limited in the trapezoidal section at a distance of (**A**) more than $\frac{1}{2}w_{T}$, (**B**) equal to $\frac{1}{2}w_{T}$, (**C**) less than $\frac{1}{2}w_{T}$. The results also show the existence of certain local secondary flows in the rectangular section of complex channel cross-section.

When fluid flows into a complex cross-sectional channel, the secondary vortices are confined in the trapezoidal section. Numerical comparison of the flow field in the cross-section of channels Design No. 3, Design No. 4, and Design No. 5 (Figure 2) indicated that the complex cross-section could result in a significant increase of the fluid velocity in the trapezoidal region rather than rectangular region. It has been demonstrated that the induced secondary flow in the trapezoidal section has the features in common with Dean flow in the normal trapezoidal channel. It can be seen from Figure 2 that there are several local vortices in the rectangular region that they are much more considerable in the channel Design No. 3 than those in the channel Design No. 4 and channel Design No. 5. This way of having a tight focusing band by confining the secondary flow in the inner part of cross-section seems more impactful than decreasing the hydraulic diameter. Based on Equation (6), we speculate that greater CFN in this kind of cross-section results in more confined Dean vortices, less local vortices (Figure 2, channel Design No. 5), and leads to well-ordered of particle focusing. Hence, there are some reasons to believe that the complex cross-sectional channel is a powerful way to limit secondary flow and altering the Dean flow pattern with more efficient high-throughput particle concentration. 

### 3.2. Effect of Inner Wall Size on Inertial Focusing. Comparison of the Focusing Behavior in an Inward and Outward Sloping Trapezoid 

**Figure 3:**
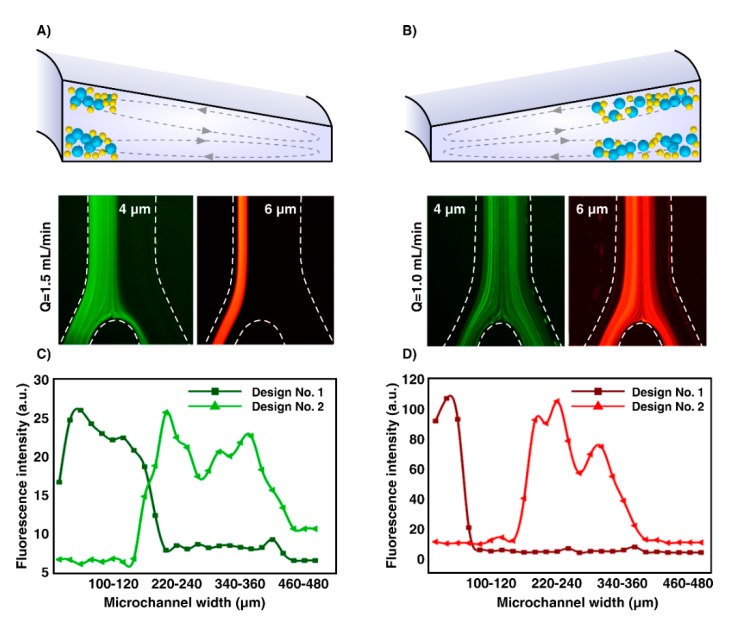
Schematic and top view experimental observation of fluorescently labeled particles indicating the inertial focusing of 4 µm and 6 µm beads in (**A**) Design No. 1 and (**B**) Design No. 2 at the optimal flow rate (1.5 mL/min). The plot of fluorescent intensity against channel width indicates the distribution of (**C**) 4 µm (green) and (**D**) 6 µm (red) particles flowing in the spiral Design No. 1 and Design No. 2 devices as a function of channel cross-sectional geometry.

To demonstrate the inertial migration of particles to the equilibrium positions near the inner microchannel wall, 4µm and 6µm particles were introduced individually into the spiral microchannel through inward sloping (Figure 3A) and outward sloping (Figure 3B) trapezoidal cross-sections. The hydraulic diameter of channels was the same, and the optimal flow rate was selected as 1.5 mL/min based on our previous studies [41]. Under the complex coupling of the inertial lift and the Dean drag forces, initially dispersed particles in channel Design No. 1 migrated and assembled into a well-ordered stream located close to the inner wall. The focusing band of 4µm particles is much thicker than that of 6µm particles, illustrating the strong dependence of the lift force on the particle diameter compared to drag force ($F_{L}\propto a_{p}^{4},\;\;F_{D}\propto a_{p}$) [42,43]. In addition, as shown in Figure 3, the fluorescence intensity of 4 µm and 6 µm plotted against microchannel width at the outlet indicates the distribution of 4 µm and 6 µm particles which is depended on the channel cross-sectional geometry. Irrespective of the particle sizes, at the outlet of Design No. 1, particles tend to form a narrow focusing band towards the inner wall, unlike the particles flowing in Design No. 2. The 6 µm particles formed a focused stream at a distance of ~90µm away from the inner wall in the top view, and 4 µm particles occupied a similar lateral position but had a wider stream width due to the smaller particle size. Particles in Design No. 2 tend to form broadbands towards the middle of the channel. From Figure 3C and D, particles in Design No. 2 concentrate in a wide stream at the distance of 120–460 µm and 110–410 µm of the inner channel wall. As a result, particles through Design No. 1 with the deeper inner wall are trapped into strong Dean vortices cross skewed toward the wall, performed at the inner side of the channel; thus, all particles were focused at the inner half of the channel. This equilibration is primarily due to the stronger lift forces. Then, the strong lateral Dean flow further modifies the equilibrium positions to one narrow stream near the inner wall because of the curvilinear nature of the spiral microchannel [12,17,44]. Seen together, the results suggest that the Design No. 1 rather than the Design No. 2 is an optimum design considered for isolating particles from its carrier fluid.

### 3.3. Effect of Channel Cross-sectional Shape. Altering the Geometry of Channel Cross-section 

Equilibrium positions of flowing particles in microchannels are highly dependent on their size [12,45]. $C_{R}$ is a design parameter to determine the focusing behavior of particles within the microchannels. For $C_{R}\geq 0.07$, the inertial lift forces dominate over the Dean drag force, and successful inertial focusing occurs [12]. Values of $C_{R}$ for 4 µm and 6 µm particles in Design No. 1 and Design No. 4 tested in this work were summarized in Table 1. The estimated $C_{R}$ of particles in two microchannels is less than the reported $C_{R}\geq 0.07$ to achieve tight focusing streams. 

**Figure 4:**
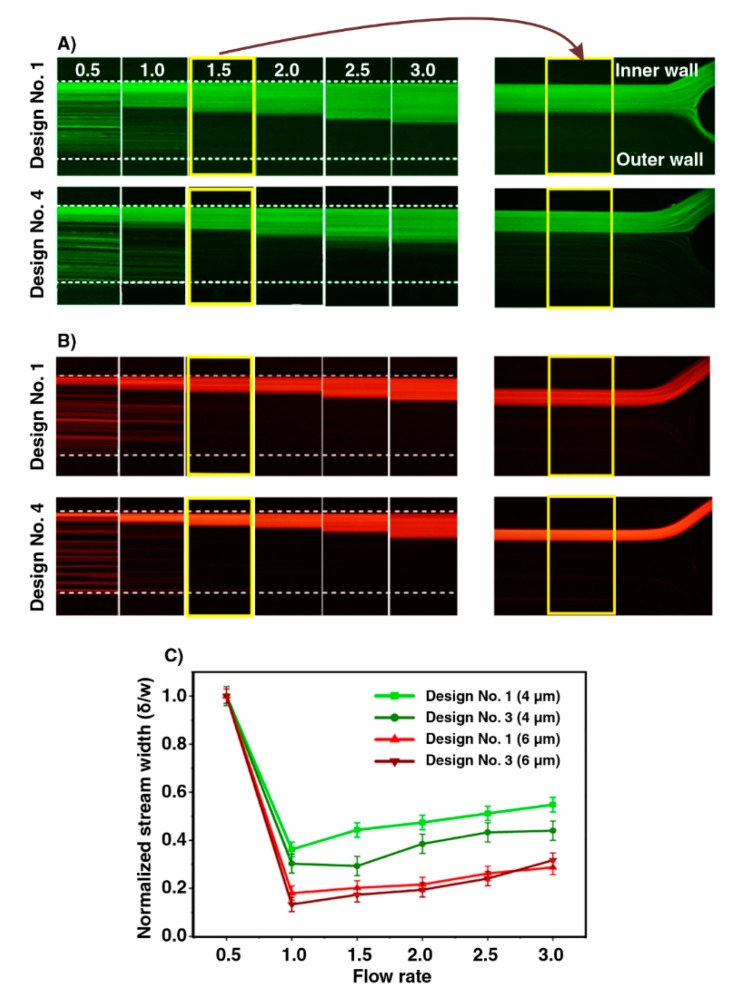
Fluorescent images illustrate the control of the particle stream width as a function of channel geometry at the flow rates ranged from 0.5 to 3.0 mL/min. (**A**) 4 µm particles through Design No. 1 and Design No. 4. (**B**) 6 µm particles within Design No. 1 and Design No. 4. Each column compares the position of focusing band at different cross-sections for different particle sizes. Particles in channel Design No. 4 migrate into well-ordered equilibrium positions under the influence of the *F_L_* and *F_D_*. (**C**) Normalized stream width (*δ* is the width of the particle stream and *w* is the width of the channel) of 4 µm and 6 µm particles in Design No. 1 and Design No. 4 was plotted as a function of sample flow rate.

Figure 4 shows the results of focusing trajectories of 4 µm and 6 µm particles in the top view flown through Design No. 1 and Design No. 4. The 4 µm particles in both microchannels migrate towards the inner wall and occupy the equilibrium positions at given flow rates ranged from 1.0 to 2.0 mL/min (Figure 4A). Increasing the flow rate further dominates the Dean drag force over the lift forces resulting in disturbance of focusing positions and de-focusing of the particle focusing bands [12]. Particles are expected to re-circulate with the fluid flow, and a complete recirculation occurred by the time particles reach the outlet. As can be seen from Figure 4B, 6 µm particles were assembled into a narrow focusing band located close to the inner wall, and they were kept focused in a single stream under wide ranges of flow rates (from 1 to 3 mL/min). Particles gradually departed from their equilibrium positions by further increasing the flow rate. Figure 4C confirms that the normalized stream width of both particle sizes in Design No. 4 is smaller than Design No. 1. 

**Figure 5:**
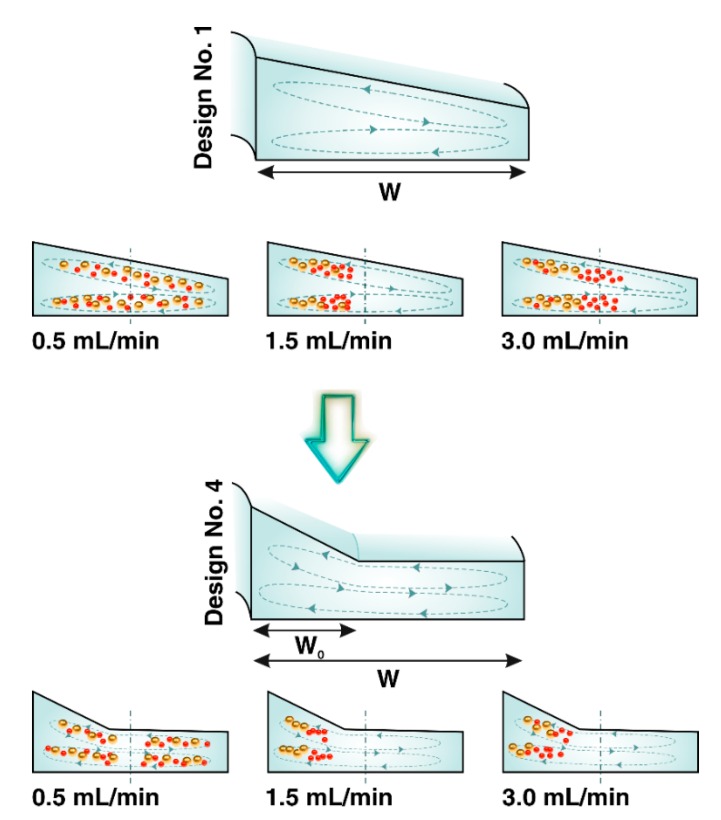
Schematic (not to scale) of trapezoidal (Design No. 1) and complex cross-section (Design No. 4) and lateral position for a differential displacement of particles at different flow rates. Schematic shows particles through Design No. 4 focused closer to the inner wall, progressively displaced away from the inner wall without de-focusing by increasing fluid velocity. This suggests that precise control of the lateral position can be achieved through a channel with a complex cross-section.

The presence of secondary flows in our spiral microchannels was confirmed from the simulation results of the flow field in the channel cross-sections (Figure 2). It is clear that Dean vortices are limited in the trapezoidal region of the complex cross-section. The Dean vortices in the trapezoidal cross-section have extended along the channel width while they are confined in the trapezoidal region of the complex cross-section. Moreover, the magnitude of the fluid velocity flowing in the trapezoidal region is more than that in the rectangular section. Combined with these results, the experimental observations illustrated that the particle stream width became thinner in Design No. 4, i.e., complex-shaped cross-section, rather than Design No. 1. Therefore, altering the geometry of cross-section from the normal into the complex has a direct result of decreasing the *D_h_* and further modification of the particle equilibrium positions, making the focusing band tighter (Figure 5). Strong Dean vortex cores are formed near the inner longer-side wall, which exerts an extremely powerful *F_D_* dominating *F_L_* pushing particles more toward the inner wall. All particles experience higher *F_D,_* and nearly 98% of the randomly dispersed particles at the inlet migrated to the inner half by the time the fluid reached the outlet.

### 3.4. Effect of CFN on Particle Focusing 

**Figure 6:**
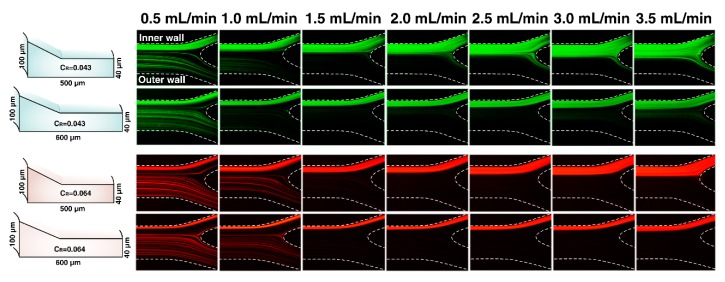
Fluorescent images of the outlet of Design No. 4 and Design No. 5 are shown. Cross-sectional views are given for comparison (**left**). Fluorescent stream images indicate the distribution of 4 µm (**green**) and 6 µm (**red**) at different flow rates.

Following the determination of the channel geometry parameter to acquire successful particle focusing, we have designed and fabricated two more complex cross-sectional channels with different CFNs. The CFN of Design No. 4 and Design No. 5 is ~4.5 and ~6, respectively (see the Supplementary Material and Figure S1). It was observed that Design No. 5 focused 4 µm and 6 µm particles in a single stream at varying flow rates from 1 to 3.5 mL/min, successfully (Figure 6). Particle focusing trajectories in both channels indicate that the particle focusing bands in Design No. 5 are much thinner than Design No. 4, compared with Martel and Toner’s results [46], which illustrate that widening the particle focusing band occurs as the overall channel width is increased. Interestingly, particles within Design No. 5 are gathered in a single narrow stream along the channel cross-section and remained in a tight focusing status at a wide ranges of flow rates from 1 to 3.5 mL/min. On the contrary, particles through Design No. 4 start to defocus by increasing flow rate above 1.5 mL/min. Asymmetry cross-section and depending on whether the CFN is large or not, the secondary flow pattern is different and influences the velocity profile of secondary flow. The confined Dean vortices in channel Design No. 4 and channel Design No. 5 were limited near the inner longer-side wall in the distance of $\frac{1}{2}W_{T}$ and $<\frac{1}{2}W_{T}$, respectively. From Figure 2, it is clear that arrows indicating the secondary flow do not follow the Dean flow pattern in the rectangular region, and they are dispersed in different directions. Moreover, the magnitude of fluid velocity in the trapezoidal region is much higher than that in the rectangular region. On the other hand, for particles satisfying $C_{R}\geq 0.07$, the inertial lift forces dominate over the Dean drag force and particles tend to focus in a single equilibrium position [12]. Under this situation, 4 µm successfully focused through a spiral channel with a hydraulic diameter of ~57 µm. Nonetheless, this gives rise in the pressure and causes difficulty or even impossibility for pumping through the channel. In comparison, Design No. 5, as a new proposed complex cross-section, could overcome this problem by having a larger hydraulic diameter (~93 µm) and $C_{R}\;$~0.043. Apart from these, separation efficiency (SE) (Equation (7)) of 4 µm fluorescent particles in high and low concentration were achieved in particles collected from inner outlet (target outlet) (Table 2).

(7) $$SE=\frac{No.\;of\;particles\;collected\;in\;the\;target\;outlet}{No.\;of\;particles\;collected\;from\;all\;outlets}$$

Utilizing channel Design No. 5 provides the continuous concentration of particles in volumes up to more than 500 times smaller than the initial input after certain recirculation, leading to the isolation and concentration of particles at extremely high throughput manner.

**Figure 7:**
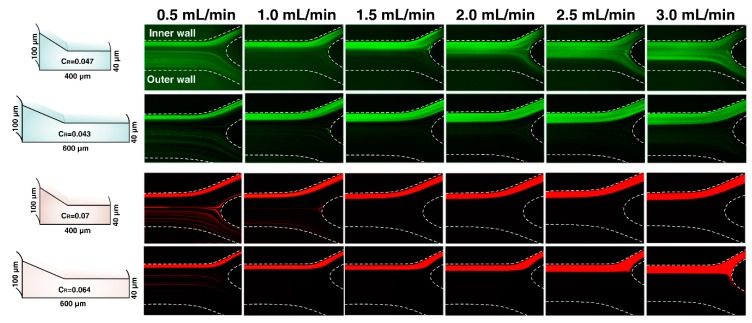
Fluorescent images of the outlet of Design No. 5 and Design No. 3 are shown. Cross-sectional views are given for comparison (**left**). Fluorescent stream images indicate the distribution of 4 µm (**green**) and 6 µm (**red**) at six different flow rates.

In the following, to investigate the influence of Dean vortices regulation on particle focusing manner, we considered Design No. 3 in larger $C_{R}$ and less CFN (4.3) than Design No. 5. Figure 7 illustrates the images extracted from experimental data of 4 µm and 6 µm particles across Design No. 3 and Design No. 5. It can be seen that the degree of focusing decreases with Re along Design No. 3, leading to the defocusing of particles. There is conformity between experimental and simulation results in existing the strong Dean flow in the trapezoidal region of complex cross-sectional shape. More importantly, the simulation results of the flow field in the cross-section of channel Design No. 3 shows the existence of local vortices in the rectangular region that influences the focusing behavior of particles. Dean vortices in channel Design No. 3 were limited in the distance of $>\frac{1}{2}W_{T}$ which increases the possibility of dislocating particles from the trapezoidal region into the rectangular region. Particles get trapped in these local secondary flows disturbing the focusing status and distribute across the channel cross-section. The results indicate that although the large $C_{R}$ guarantees the tight focusing bands of particles, our experiments have shown that there is an optimal amount of CFN = 6 (Design No.5). As a result, $C_{R}$ has to be calculated meticulously, which is strongly dependent on channel cross-sectional geometry rather than hydraulic diameter. It altered to a value of less than 0.07 through our complex cross-sections. 

According to our results, changing of parameters such as decreasing the amount of CR and increasing the amount CFN in these proposed complex cross-sections leads to isolating 4 µm and 6 µm particles. CFN number is a parameter that depends on the channel cross-section, and it changes by altering the channel height, overall channel width, and slant of trapezoidal region proportional to its width. Therefore, there is a possibility to isolate different particle sizes by manipulating this number beside other parameters. Also, new modifications can be applied to confine Dean vortices in addition to decreasing CR. 

## 4. Conclusions

In summary, we have introduced a novel spiral microchannel with complex cross-section for the continuous concentration of particles/cells. We have demonstrated, for the first time, that incorporating trapezoidal and rectangular shapes into one shape called complex cross-section confines the Dean vortices in the trapezoidal region resulting in efficient focusing of particles with high throughput. The device altered the threshold of $C_{R}$ to a number less than 0.07, achieving a well-ordered particle focusing. All in all, there has been no spiral channel observed with C_R_ ~0.043 to focus particles in narrow focusing bands at high flow rates and with high fidelity. In addition, we have introduced another new parameter, CFN, for designing complex cross-sectional microchannels combination of trapezoidal and rectangular shapes. CFN, in this kind of cross-sections, determines the degree of particle focusing and the limited amount of Dean vortices. Results showed that the Dean vortices confined more in channel Design No. 5 resulted in powerful dean vortices cores in the trapezoidal region. Particles were trapped in the distance of $<\frac{1}{2}W_{T}$ and remained focused at high flow rates without dislocating in local vortices located in the rectangular region. The cross-section with CFN = 6 has been proven to be able to conduct a continuous particle concentration with high efficiency (~98%). In addition, the output volume was 500 times smaller than the initial input accomplished through channel Design No.5. Finally, for future work, we suggest designing microchannels with higher CFN than 6 to compare particle focusing in complex cross-sections, a combination of rectangular and trapezoidal shapes. In addition, this research predicts that there are possibilities for introducing new parameters to control particle focusing for the efficient separation of particles/cells. 

## Figures and Tables

**Table 1:** Summary of confinement ratio for 4 µm and 6 µm particles in varying hydraulic diameter.

Particle Diameter (µm)	Design	Hydraulic Diameter (*D_h_*) (mm)	Confinement Ratio (*CR*)
4	Design No. 1	0.122	0.032
	Design No. 4	0.093	0.042
6	Design No. 1	0.122	0.049
	Design No. 4	0.093	0.064

**Table 2:** The separation efficiency of 4 µm fluorescent particles in a high and low concentration.

	High Concentration		Low Concentration	
	Per mL	Total	Per mL	Total
**Inner outlet (target outlet)**	822,500	2,673,125	4000	13,000
**Outer outlet**	42,500	74,375	66.66	166.655
**Separation efficiency**	97.29%		98.73%	

## Supplementary Materials

The following are available online at https://www.mdpi.com/2072-666X/11/4/440/s1, Figure S1: Schematic of complex cross-sectional channels illustrating dimensional characteristic.
