# Supplementary material for: High-Throughput Particle Concentration Using Complex Cross-Section Microchannels
Source: Micromachines (Basel). 2020 Apr 22;11(4):440. doi: 10.3390/mi11040440 (PMC7231362; doi:10.3390/mi11040440)
Supplement: Supplementary file 1 [file micromachines-11-00440-s001.pdf]

micromachines

MDPI

Supplementary Materials: High-Throughput Particle Concentration Using Complex Cross-Section Microchannels

Asma Mihandoust [1], Sajad Razavi Bazaz [2], Nahid Maleki-Jirsaraei [1], Majid Alizadeh [3], Robert A. Taylor [4] and Majid Ebrahimi Warkiani [2,5,*]

[1] Complex Systems Laboratory, School of Physics-Chemistry, Department of Physics, Alzahra University, Tehran 1993893973, Iran; a.mihandoust@alzahra.ac.ir (A.M.); maleki@alzahra.ac.ir (N.M.-J.)
[2] School of Biomedical Engineering, University of Technology Sydney, Sydney, NSW 2007, Australia; sajad.razavibazaz@student.uts.edu.au
[3] School of Paramedicine, Ilam University of Medical Science, Ilam 6939177143, Iran; alizadeh_majid@ymail.com
[4] School of Mechanical and Manufacturing Engineering, University of New South Wales, Sydney, NSW 2052, Australia; robert.taylor@unsw.edu.au
[5] Institute of Molecular Medicine, Sechenov University, Moscow 119991, Russia
* Correspondence: majid.warkiani@uts.edu.au

Received: 24 March 2020; Accepted: 20 April 2020; Published: date

Calculation of Focusing Number of Complex cross-section (FNC):

The FNC number of the spiral microchannel can be obtained as follows:

$$FNC = \cot\alpha \, . \frac{W_R}{W_T} \quad \text{(S-1)}$$

Where α is the angle between sloping side and the horizontal line, w_R is the devoted width to the trapezoidal shape, and w_T is the devoted width to the rectangular shape. The value of FNC affects the threshold of sample flow rates to start the de-focusing of particles from equilibrium positions and moves to the higher flow rates within microchannel with large amount of FNC.

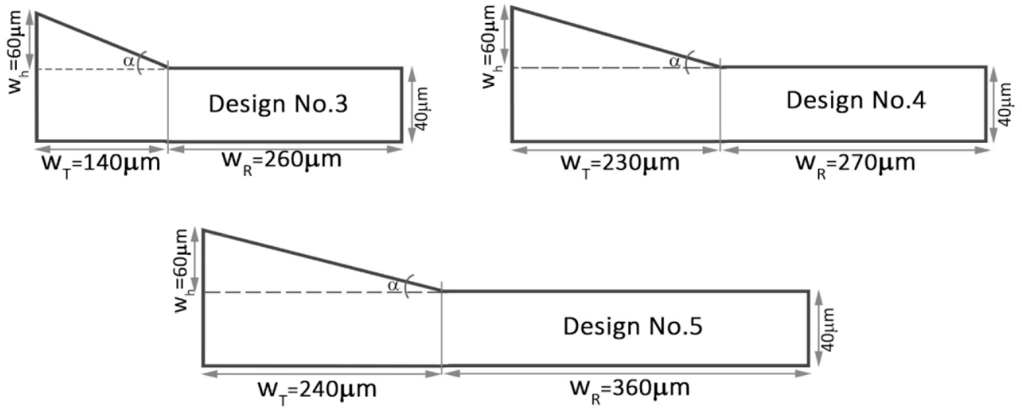

Figure S1. Schematic of complex cross-sectional channels illustrating dimensional characteristic.

The FNC number of complex cross-sectional channels can be estimated as follow:
Channel Design No. 3:

$$FNC = \cot\alpha \, . \frac{W_R}{W_T} \quad \text{(S-2)}$$

$$FNC = \frac{140}{60} . \frac{260}{140} = 4.3 \quad \text{(S-3)}$$

Channel Design No. 4:

$$FNC = \cot\alpha . \frac{W_R}{W_T} \quad \text{(S-4)}$$

$$FNC = \frac{230}{60} . \frac{270}{230} = 4.5 \quad \text{(S-5)}$$

Channel Design No. 5:

$$FNC = \cot\alpha . \frac{W_R}{W_T} \quad \text{(S-6)}$$

$$FNC = \frac{240}{60} . \frac{360}{240} = 6 \quad \text{(S-7)}$$

The FNC number can be written as

$$FNC = \cot\alpha . \frac{W_R}{W_T} = \frac{W_T}{W_h} . \frac{W_R}{W_T} = \frac{W_R}{W_h} \quad \text{(S-8)}$$

Therefore, regarding (S-8), the FNC can be obtained as

$$FNC = \frac{W_R}{W_h} \quad \text{(S-9)}$$

The above formula can be considered for complex cross sectional channels, combination of trapezoidal and rectangular sections.
